# Complications of Alcohol Withdrawal: Pathophysiological Insights

**Published:** 1998

**Authors:** Louis A. Trevisan, Nashaat Boutros, Ismene L. Petrakis, John H. Krystal

**Affiliations:** Louis A. Trevisan, M.D., is an assistant clinical professor, Nashaat Boutros, M.D., is an associate professor, Ismene L. Petrakis, M.D., is an assistant professor, and John H. Krystal, M.D., is an associate professor in psychiatry at the Department of Psychiatry, Yale University, New Haven, Connecticut

**Keywords:** AOD withdrawal syndrome, disease severity, disease complication, AODR (alcohol and other drug related) seizure, delirium tremens, Wernicke Korsakoff psychosis, anxiety state, emotional and psychiatric depression, sleep disorder, mood and affect disturbance, heart disorder, acute AODE (alcohol and other drug effects), AODD (alcohol and other drug dependence) relapse, GABA receptors, glutamate receptors, sex hormones, drug therapy, AOD abstinence, literature review

## Abstract

Disease processes or events that accompany acute alcohol withdrawal (AW) can cause significant illness and death. Some patients experience seizures, which may increase in severity with subsequent AW episodes. Another potential AW complication is delirium tremens, characterized by hallucinations, mental confusion, and disorientation. Cognitive impairment and delirium may lead to a chronic memory disorder (i.e., Wernicke-Korsakoff syndrome). Psychiatric problems associated with withdrawal include anxiety, depression, and sleep disturbance. In addition, alterations in physiology, mood, and behavior may persist after acute withdrawal has subsided, motivating relapse to heavy drinking. Recent advances in neurobiology may support the development of improved medications to decrease the risk of AW complications and support long-term sobriety.

Abrupt reduction or total cessation of long-term alcohol consumption produces a well-defined cluster of symptoms called acute alcohol withdrawal (AW). Although some patients experience relatively mild withdrawal symptoms, disease processes or events that accompany AW can cause significant illness and death. After acute withdrawal has subsided, a poorly defined syndrome of protracted withdrawal may ensue. The persistent alterations in physiology, mood, and behavior associated with protracted withdrawal may motivate the patient to relapse to heavy drinking. This article describes the acute withdrawal syndrome and its complications, including seizures, delirium tremens, Wernicke-Korsakoff syndrome, neuropsychiatric disturbances, and cardiovascular complications as well as the protracted withdrawal syndrome. Recent findings are discussed regarding the alcohol-induced alterations of nervous system function that underlie these syndromes and their implications for the treatment of withdrawal.

## Acute Alcohol Withdrawal Syndrome

Alcohol withdrawal is a distinctive clinical syndrome with potentially serious consequences (see table) (American Psychiatric Association 1994). Symptoms begin as early as 6 hours after the initial decline from peak intoxication. Initial symptoms include tremor, anxiety, insomnia, restlessness, and nausea. Particularly in mildly alcohol-dependent persons, these symptoms may comprise the entire syndrome and may subside without treatment after a few days. More serious withdrawal symptoms occur in approximately 10 percent of patients. These symptoms include a low-grade fever, rapid breathing, tremor, and profuse sweating. The time course of withdrawal is outlined in the figure on p. 63. Seizures may occur in more than 5 percent of untreated patients in acute alcohol withdrawal. Another severe complication is delirium tremens (DT’s), which is characterized by hallucinations, mental confusion, and disorientation. The mortality rate among patients exhibiting DT’s is 5 to 25 percent.

## Seizures

Withdrawal seizures usually consist of generalized convulsions alternating with spasmodic muscular contractions (i.e., tonic-clonic seizures). Seizures that begin locally (e.g., with twitching of a limb) suggest the presence of a co-occurring disorder, which should be fully investigated.

More than 90 percent of alcohol withdrawal seizures occur within 48 hours after the patient stops drinking. Fewer than 3 percent of such seizures may occur 5 to 20 days after the last drink (Victor and Brausch 1967). Clinical data suggest that the likelihood of having withdrawal seizures, as well as the severity of those seizures, increases with the number of past withdrawals. The correlation between the number of alcohol detoxifications and the development of alcohol withdrawal complications, including seizures, has been ascribed to cumulative long-term changes in brain excitability (i.e., the “kindling” hypothesis) (Ballenger and Post 1978; Brown et al. 1988). (For further discussion on kindling, see the article by Becker, pp. 25–33.)

## Delirium Tremens

DT’s are a serious manifestation of alcohol dependence that develops 1 to 4 days after the onset of acute alcohol withdrawal in persons who have been drinking excessively for years. Signs of DT’s include extreme hyperactivity of the autonomic nervous system,^1^ along with hallucinations. Women experiencing DT’s appear to exhibit autonomic symptoms less frequently than men. Co-occurring medical problems may obscure the diagnosis and treatment of DT’s or worsen the outcome. Such medical problems include altered blood chemistry, certain infections, and Wernicke’s syndrome (see the following section for a discussion on Wernicke’s syndrome) (Saitz 1995). Death may occur in up to 5 percent of patients with DT’s. The risk of death is reduced, however, in patients receiving adequate medication and medical support.

Alcoholics who are awaiting surgical or medical treatment often exhibit DT’s when their alcohol consumption is abruptly interrupted by hospitalization. Therefore, hospital staff must remain vigilant for signs and symptoms of alcohol withdrawal, even in patients not known to be alcoholic. In addition, clinicians must learn to differentiate DT’s from other possible causes of delirium (Alvi and Gonzalez 1995).

**Table:** Diagnostic Criteria for Alcohol Withdrawal^1^

Cessation of or reduction in alcohol use that has been heavy or prolonged. Two or more of the following symptoms have developed within hours to a few days after criterion 1: Autonomic hyperactivity (for example, sweating or pulse greater than 100 beats per minute) Increased hand tremor Insomnia Transient visual, tactile, auditory hallucinations or illusions Nausea or vomiting Excessive, purposeless physical activity (i.e, psychomotor agitation) Anxiety Grand mal seizures. The symptoms in criterion 2 cause clinically significant distress or impairment in social, occupational, or other important areas of functioning. The symptoms are not attributable to a general medical condition and are not better accounted for by another mental disorder.

1 As defined in the *Diagnostic and Statistical Manual of Mental Disorders, Fourth Edition* (American Psychiatric Association 1994).

The prediction of complicated alcohol withdrawal is an important part of alcoholism treatment to ensure that appropriate therapies may be planned in advance. Risk factors for prolonged or complicated alcohol withdrawal include lifetime or current long duration of alcohol consumption, lifetime prior detoxifications, prior seizures, prior episodes of DT’s, and current intense craving for alcohol (Saitz 1995). Certain clinical and biochemical findings have been associated with high risk for the development of DT’s, including specific alterations of blood chemistry; elevated liver enzymes; and certain nervous system disturbances, including muscular incoordination (Wetterling et al. 1994).

## Wernicke-Korsakoff Syndrome

The combination of Wernicke’s and Korsakoff’s syndromes is not a complication of AW but rather of a nutritional deficiency. Nevertheless, the syndromes usually occur during AW. Wernicke’s syndrome is a disorder of the nervous system caused by thiamine deficiency, and alcoholics account for most cases in the Western world (Victor et al. 1989). The syndrome is characterized by severe cognitive impairment and delirium, abnormal gait (i.e., ataxia), and paralysis of certain eye muscles (reviewed in Charness 1993). A majority of patients are profoundly disoriented, indifferent, and inattentive; some exhibit an agitated delirium related to alcohol withdrawal. Ocular signs improve within hours to days; ataxia and confusion improve within days to weeks. A majority of patients are left with an abnormal gaze, persistent ataxia, and a potentially disabling memory disorder known as Korsakoff’s syndrome. Although fewer than 5 percent of patients initially exhibit a depressed level of consciousness, the course in untreated patients may progress through stupor, coma, and death. Nutritional status should be closely monitored during treatment of acute AW to prevent Wernicke-Korsakoff syndrome (for more details, see the article by Myrick and Anton, pp. 38–43).

**Figure:**
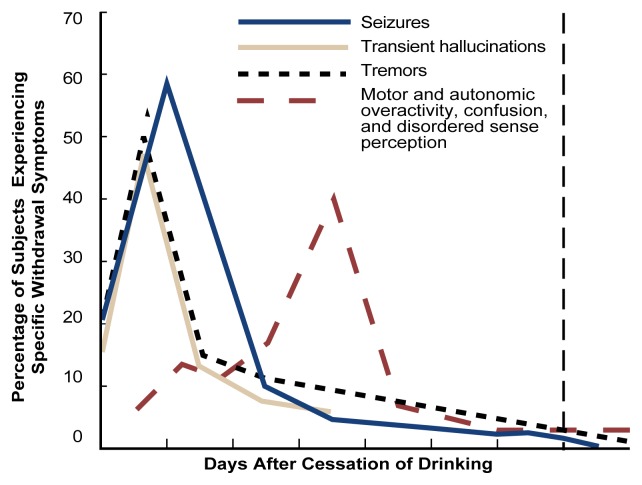
The relationship between cessation of drinking and the onset of tremors, hallucinations, seizures, and delirium tremens. SOURCE: Adapted from Victor and Adams 1953.

Approximately 80 percent of alcoholic patients recovering from Wernicke’s syndrome exhibit the selective memory disturbance of Korsakoff’s syndrome (Victor et al. 1989). Symptoms of Korsakoff’s syndrome include severe amnesia for past events, along with impaired ability to commit current experience to memory. The patient often recites imaginary experiences to fill gaps in his or her memory. Although the patient may be apathetic, intellectual abilities other than memory are relatively preserved (Charness 1993). Korsakoff’s syndrome can occur in the absence of alcohol use; however, the disease rarely follows Wernicke’s syndrome in nonalcoholics. This observation has lead to speculation that the neurotoxicity of alcohol is an important contributing factor in the memory disorders of alcoholics (Charness 1993).

## Disturbances of Mood, Thought, and Perception

Withdrawing alcoholics exhibit psychiatric difficulties that may be related to the process of withdrawal itself or to co-occurring conditions. The major psychiatric problems associated with acute and protracted withdrawal are anxiety, depression, and sleep disturbance. Less frequently, psychotic symptoms, including delusions and hallucinations, may be associated with withdrawal (Smith 1995).

### Anxiety

Anxiety disorders are manifested by extreme fear and anxiety, accompanied by heart palpitations; shallow, rapid breathing (i.e., hyperventilation); sweating; and dizziness. Alcohol has antianxiety properties that promote its use to self-medicate anxiety (George et al. 1990*a*,*b*). However, prolonged alcohol use—and especially acute AW states—can increase anxiety levels. Marked signs of anxiety commonly appear between 12 and 48 hours after cessation of alcohol consumption (Peyser 1982).

Hyperventilation may occur during acute withdrawal, leading to disturbed blood chemistry and resulting in symptoms that may be indistinguishable from those that occur in anxiety disorders (Kushner et al. 1990). Some researchers have hypothesized that repeated AW may predispose alcoholics to certain anxiety disorders through the process of kindling (see the article by Becker, p. 25–33) (Lepola 1994).

### Depression

Depressive symptoms often are observed in patients who are intoxicated or undergoing alcohol detoxification. As many as 15 percent of alcoholics are at risk for death by suicide, and recent consumption of alcohol appears to increase the danger of a fatal outcome from self-harm (Madden 1993). This finding may be attributable to the release of behavioral inhibition associated with alcohol intoxication or with the depressive feeling states that accompany the decline from peak intoxication. Depressive disorders commonly emerge during AW (Madden 1993); in addition to the depressive feeling states associated with alcohol consumption and withdrawal, the social, psychological, and physical problems associated with alcoholism may contribute to the development of depressive disorders.

### Sleep Disturbances

Sleep disturbances—including frequent awakening, restless sleep, insomnia, and night terrors—are among the most common complaints of alcoholics (Smith 1995). Sleep problems persist into AW, with pronounced insomnia and marked sleep fragmentation (Le Bon et al. 1997). In addition, alcoholics show increased incidence of interrupted breathing during sleep compared with the general population. These sleep disturbances can cause daytime drowsiness, reducing the efficiency of performance of daytime tasks and increasing the risk of car crashes (Aldrich in press).

### Hallucinations and Perceptual Disturbance

Visual, auditory, and tactile hallucinations are frequently experienced in acute, complicated AW or DT’s. Hallucinations that are not connected with DT’s occur in 3 to 10 percent of patients during severe AW from 12 hours to 7 days after cessation or reduction of alcohol consumption (Platz et al. 1995).

In one study, 10 percent of 532 male patients admitted to a Veterans Affairs Hospital for AW developed hallucinations (Tsuang et al. 1994). Patients who hallucinated tended to be younger at the onset of their alcohol problems, consumed more alcohol per drinking occasion, developed more alcohol-related life problems, and had higher rates of other drug use than patients who did not hallucinate.

## Cardiovascular Complications

The heart is a major site of alcohol-induced organ damage, including disturbances of heartbeat rhythm (Smith 1995). For example, the “holiday heart syndrome” consists of episodes of abnormal cardiac rhythms following a bout of drinking (Smith 1995). Because arrhythmia generally occurs after a binge, rather than during intoxication, AW may be a contributing factor to the occurrence of alcohol-related arrhythmia (Smith 1995). Further study is required to elucidate the possible connection between AW and increased sudden cardiac death.

## Protracted Withdrawal Syndrome

Data appear to indicate that a protracted withdrawal syndrome (PWS) may develop following AW and may persist for at least 1 year. Some manifestations of PWS include symptoms associated with AW that persist beyond their typical time course. These symptoms include tremor; sleep disruption; anxiety; depressive symptoms; and increased breathing rate, body temperature, blood pressure, and pulse (Alling et al. 1982; Schuckit et al. 1991). Other symptoms of PWS appear to *oppose* symptoms of AW. These symptoms of PWS include decreased energy, lassitude, and decreased overall metabolism (Satel et al. 1993).

The significance of this cluster of symptoms has been debated (Satel et al. 1993). For example, PWS could reflect the brain’s slow recovery from the reversible nerve cell damage common in alcoholism. Clinically, the symptoms of PWS are important, because they may predispose abstinent alcoholics to relapse in an attempt to alleviate the symptoms (Satel et al. 1993).

## Neurobiology of Alcohol Withdrawal

Alcohol affects the way in which nerve cells communicate. For example, alcohol’s sedating effect is related to altered function of specific receptors in the brain. Receptors are specialized proteins on the surface of nerve cells that receive chemical signals from other cells. These signals are generally conveyed by chemical messengers released by nearby nerve cells (i.e., neurotransmitters). With long-term alcohol consumption, receptors affected by alcohol undergo adaptive changes in an attempt to maintain normal function. When alcohol consumption ceases, these changes are no longer adaptive and may contribute to the phenomena associated with AW. Two important brain communication systems affected by alcohol involve the neurotransmitters gamma-aminobutyric acid (GABA) and glutamate.

### The GABA System

GABA is an inhibitory neurotransmitter that helps to regulate brain function by rendering nerve cells less sensitive to further signaling. Single doses of alcohol facilitate the inhibitory function of the GABA_A_ receptor, contributing to alcohol’s intoxicating effects (Suzdak et al. 1986). During withdrawal, brain GABA levels fall below normal and GABA activity declines (Petty et al. 1993). In addition, the sensitivity of GABA_A_ receptors to chemical signals also may be reduced in recently detoxified alcoholic patients (Gilman et al. 1996). The combination of reduced brain GABA levels and GABA_A_-receptor sensitivity may be considered an adaptation to the presence of alcohol. In the absence of alcohol, the resulting decrease in inhibitory function may contribute to symptoms of nervous system hyperactivity associated with both acute and protracted AW.

### The Glutamate System

The major excitatory neurotransmitter in the brain is glutamate, which communicates with three major subtypes of glutamate receptors. Among these, the *N*-methyl-d-aspartate (NMDA) receptor plays a role in memory, learning, and the generation of seizures (Finn and Crabbe 1997). Alcohol inhibits the excitatory function of the NMDA receptor in laboratory studies at concentrations associated with mild to moderate alcohol intoxication in humans (Lovinger et al. 1989; Simson et al. 1991). As with the increased inhibitory function of the GABA_A_ receptor, the decreased excitatory function of the NMDA receptor is consistent with alcohol’s general sedative effect. Long-term alcohol administration produces an adaptive increase in the function of NMDA receptors (Trevisan et al. 1994; Danysz et al. 1992).

Acute AW activates glutamate systems (Tsai et al. 1995). In turn, AW seizures are associated with increased NMDA receptor function (Grant et al. 1990). Persistent alterations in NMDA receptor function may potentiate the neurotoxic and seizure-inducing effects of increased glutamate release during withdrawal (Tsai et al. 1995).

## Reproductive Hormones and Alcohol Withdrawal

Declines in the levels of neurosteroids may contribute to AW. Neurosteroids are substances involved in the metabolism of reproductive hormones that also have potent and specific effects on various functions of the brain. Certain neurosteroids modulate the function of the GABA_A_ receptor (Paul and Purdy 1992; Devaud et al. 1996); plasma levels of these neurosteroids are decreased during AW (Romeo et al. 1996). Because decreases in neurosteroids may contribute to AW symptoms, these compounds may have potential as medications for alleviating withdrawal (Devaud et al. 1996).

Ruusa and Bergman (1996) investigated the role of the male reproductive hormone testosterone on withdrawal symptoms. Long-term alcohol consumption causes failure of the reproductive system in men. In addition, testosterone levels decrease during alcohol consumption and increase after withdrawal. Low levels of testosterone during AW are associated with psychological symptoms, such as indecision, excessive worrying, fatigability, and lassitude. Ruusa and Bergman (1996) suggest that testosterone be administered during detoxification to determine whether a causal relationship exists.

## Antiseizure Medications

For many years, seizures and other symptoms of AW have been treated with a class of sedating medications called benzodiazepines (e.g., Valium^®^). Several studies have demonstrated that the antiseizure medications carbamezapine (Tegretol^®^) and valproic acid (Depakene^®^) are as effective as benzodiazepines for this purpose (Stuppaeck et al. 1992; Lambie et al. 1980; Wilbur and Kulik 1981). Moreover, unlike the benzodiazepines, these antiseizure medications are not potential drugs of abuse.

Alcohol withdrawal seizures and PWS have been linked to both GABA and NMDA dysregulation. Although the mechanisms of action of carbamezapine and valproic acid are not entirely understood, both medications appear to increase GABA levels in the brain in patients with seizure disorders (Petroff et al. 1995). In addition, valproic acid at therapeutic levels appears to be effective at inhibiting seizures induced by the stimulatory effect of NMDA receptors (Czuczwar et al. 1985).

Laboratory studies suggest that valproic acid may inhibit GABA metabolism and activate GABA synthesis (Fawcett 1989). In addition, data indicate that carbamezapine decreases the flow of glutamate into slices of the hippocampus, a part of the brain involved in seizures (Olpe et al. 1985). Therefore carbamezapine and valproic acid prevent alcohol withdrawal seizures and kindling.

The antianxiety and mood-stabilizing actions of these anticonvulsants may enhance their efficacy in treating withdrawal symptoms. These actions also may help relieve the constellation of symptoms associated with PWS, perhaps resulting in fewer and more mild relapses during the period following acute withdrawal.

## Summary

AW and its complications are among the most visible consequences of alcoholism. Those syndromes arise directly from adaptations made within nerve cell communication systems that are targets of alcohol in the brain. Among its actions, alcohol acutely facilitates the activity of GABA_A_ receptor function and blocks NMDA receptor activity. The adaptations within these systems contribute to withdrawal-related symptoms, seizures, and neurotoxicity. Repeated AW episodes appear to increase the risk of future AW seizures.

Acute withdrawal symptoms and complications, including seizures, hallucinations, and DT’s, represent medical emergencies. Some complications, including Wernicke-Korsakoff syndrome, may be permanently disabling. In addition, the distress associated with acute and protracted withdrawal presents an ongoing motivation to relapse to alcohol use in recently detoxified patients. Thus, the early stages of sobriety represent a period of risk at many levels.

Available treatments suppress many symptoms and complications of AW. Consequently, greater emphasis may now be placed on developing strategies to facilitate long-term sobriety. An important step in this direction may be the development of medications that lack the addiction potential of the benzodiazepines. The antiseizure medications meet these criteria and have the added capacity to suppress kindling.

AW represents a period of significant clinical risk that requires attentive medical management. However, AW also provides an opportunity to initiate treatments that may lead to extended sobriety. As such, it is a critical component of the long-term treatment strategy for every patient with alcoholism.

## Footnotes

1 The autonomic nervous system is a division of the nervous system that helps manage the body’s response to stress.
